# Terahertz and Photoelectron Emission from Nanoporous Gold Films on Semiconductors

**DOI:** 10.3390/nano9030419

**Published:** 2019-03-12

**Authors:** Junyi Nan, Min Li, Ling Zhang, Shuai Yuan, Boqu He, Heping Zeng

**Affiliations:** 1 State key laboratory of Precision Spectroscopy, East China Normal University, Shanghai 200062, China; 52162099006@stu.ecnu.edu.cn (J.N.); boquhe@foxmail.com (B.H.); 2 Shanghai Key Lab of Modern Optical System, School of Optical-Electrical and Computing Engineering, University of Shanghai for Science and Technology, Shanghai 200093, China; minli1220@usst.edu.cn (M.L.); lzhang@usst.edu.cn (L.Z.); syuan@usst.edu.cn (S.Y.)

**Keywords:** nanoporous gold, terahertz radiation, localized surface plasmons, optical rectification

## Abstract

Efficient terahertz and photoelectron emission were observed from nano-porous gold (NPG) films deposited on an intrinsic gallium arsenide (GaAs) semiconductor substrate stimulated by femtosecond laser with pulse width of 60 fs. Time-domain THz emission and reflection spectroscopy confirmed that the free charges accelerated by irradiated femtosecond laser pulses transferred from the NPG films into the GaAs substrates. Accordingly, charges accumulation was reduced in the NPG films, resulting in a stronger emission of THz pulse than that from NPG films deposited on SiO_2_ substrate. Charges injected into the GaAs substrate enforced an observable decrease of the THz refractive index proportional to the intensity of incident light. In comparison, for NPG deposited on glass substrates, laser induced free charges were accumulated in the NPG films, and femtosecond laser pulses irradiating on the NPG films made no changes of the THz refractive index of the glass substrates.

## 1. Introduction

Much effort has been devoted to developing compact and efficient terahertz (THz) sources, and especially with the development of ultrafast nonlinear optics and material science, THz technology has enabled intriguing applications in spectroscopy, imaging and sensing. Many materials like metals, liquids and gases have been applied to generate broadband and high-energy THz pulses driven by high-intensity ultrafast lasers [1,2,3,4]. In particular, metallic thin films have been used for strong field THz generation [1,5,6,7], THz waveguides [8], and THz absorbers [9]. As demonstrated by Hilton et al. [10] and Kadlec et al. [11], THz generation from metallic films is ascribed to optical rectification, which is a second-order nonlinear process. Thus, the emitted THz energy is proportional to the intensity of the irradiating laser. Surprisingly, weak THz emission was observed from ultrathin gold films with thickness of 100 nm, as reported by Kadlec’s subsequent work [12]. Based on their investigation, Kadlec suggested that accumulated charges near the metal–glass interface formed electric field opposite to the initial current at the origin of THz generation, which eventually damped optical rectification for THz emission. The challenge to generate THz from ultrathin metallic films less than 100 nm requires novel mechanism to reduce deleterious accumulation of charges on the film surfaces. Surface-plasmon excitation on a glass grating coated with 30-nm gold films [13], and metamaterials composed of split-ring resonators (SRRs) in 40-nm gold films [14] were applied to generate efficient THz emission, wherein photoelectron accelerations and accordingly optical rectifications were engineered by surface-plasmon structures. Nevertheless, photoelectron emissions from the metallic surface are intrinsically not affected and there still exists inhomogeneous charge accumulation on the metallic nano-films.

In this paper, we report on experimental demonstration of efficient terahertz emission from nano-porous gold (NPG) films deposited on an intrinsic GaAs substrate with electron mobility of ~5300 cm^2^/V/s. Time-domain THz emission and reflection spectroscopic measurements confirmed that photoelectrons were injected from NPG/GaAs interface to the GaAs substrate, removing charge accumulation on the opposite surface of the NPG films. Intriguingly, free charges accelerated by the irradiated femtosecond laser pulses in the NPG films were altered on the NPG/GaAs interface and transferred from the NPG films into the GaAs substrate. As a result, a stronger emission of THz pulse than that from NPG films deposited on SiO_2_ substrate was observed. The photo-electron injection changed the refractive index of the GaAs substrate in the THz region, which was directly monitored by measuring the reflection of the THz probe pulses from the GaAs substrate under the circumstance that the deposited NPG films was irradiated by femtosecond laser pulses. As a contrast, for NPG deposited on a glass substrate, femtosecond laser induced free charges were accumulated in the NPG films, no photoelectron injection was observed on the NPG/glass interface, and accordingly, no change of the THz refractive index of the glass substrate was observed under femtosecond laser irradiating on the NPG films. In this case, free charges were accumulated on the NPG films, restricting efficient terahertz generation. Our work could help to understand the role of photoelectrons excited by ultrafast laser pulses in THz generation from nano-scale metallic films, and facilitate possible design of strong THz emitters with NPG films deposited GaAs.

## 2. Materials and Methods

**Figure 1:**
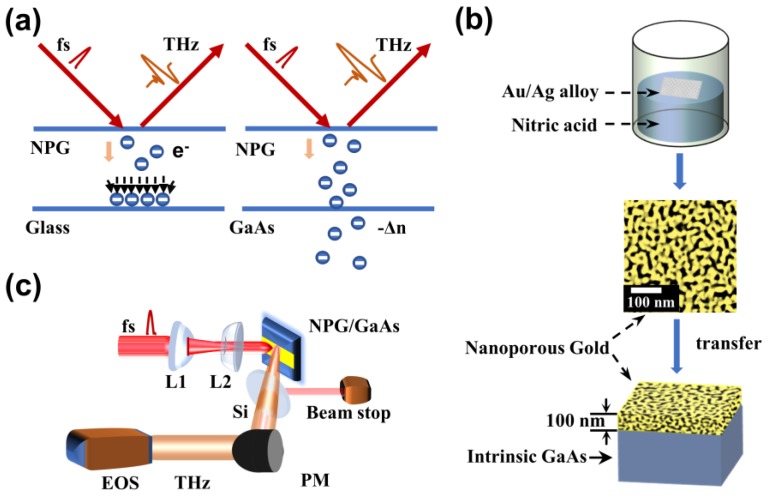
The schematic diagram of enhanced terahertz radiation and setups in our experiment. (**a**) Different processes of free charges were influenced by femtosecond laser between nano-porous gold (NPG)/glass and NPG/GaAs. Laser beam was irradiated on the surface of the NPG films, which were deposited separately on the glass slide and GaAs; (**b**) The synthesis of NPG/GaAs. The surface morphology of the NPG films was imaged by scanning electron microscope (SEM; JEOL JIB-4600F, Japan); (**c**) Schematic diagram of the experimental setup for detection of THz radiation: EOS, electro-optic sampling; PM, parabolic mirror; THz, terahertz radiation; L1, convex lens; L2, concave lens.

THz emission spectroscopy was implemented to characterize the femtosecond laser pulses induced free charges and their dynamics within the NPG films and substrates. Figure 1a shows the detected THz emission from NPG films deposited on different substrates. The NPG films in our experiments were fabricated according to the processes schematically shown in Figure 1b. NPG films were prepared from an Au_25_Ag_75_ (atom %) alloy films with thickness of 100 nm which were immersed in saturated nitric acid for 5 min. Nitric acid selectively etched silver atoms away from the alloy films, leaving three-dimensional NPG films floating on the surface of the liquid [15]. On this condition, we fabricated NPG films with various pore size of 15 nm in average diameter. The as-prepared NPG films were then physically deposited to a clean and polished 350-μm-thick intrinsic GaAs crystal and 1.5-mm-thick glass slide, respectively. After drying in air to strengthen bonding between the NPG films and the substrates, the as-prepared samples were used directly in our experiments. In what follows, the surface with the NPG films was denoted as the front surface.

As shown in Figure 1c, a Ti:Sapphire femtosecond laser system (COHERENT Inc., Santa Clara, USA) based on chirped-pulse-amplification was employed to provide 800-nm laser pulses with 60-fs pulse duration and 1 kHz repetition rate. The output laser pulse from a beam-splitter was divided into a strong part as the pump pulse and a weak part as the probe one. The laser beam is transformed by a convex lens (focus length 250 mm) and a concave lens (focus length −75 mm) in order to obtain a smaller beam size. The beam area was ~9 mm^2^. The horizontally polarized laser beam was irradiated onto the front surface of the sample with 45° incident angle. The generated THz radiation was collected by a pair of off-axis parabolic mirrors at a distance of 5 cm from the sample in the direction of specular reflection. THz radiation was detected in an optimized condition by electro-optical sampling using a 1.5-mm-thick ZnTe <110> crystal.

## 3. Results and discussion

**Figure 2:**
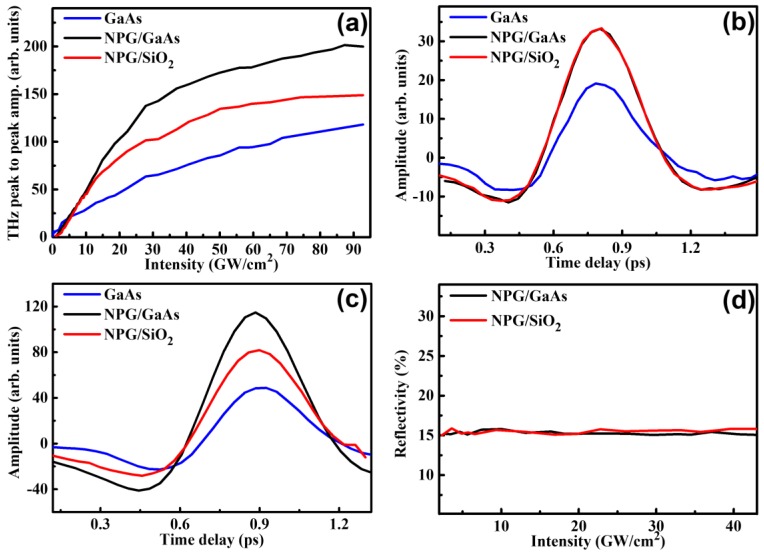
(**a**) Measured peak-to-peak amplitudes of far-field THz radiation plotted as a function of optical intensity of the incident femtosecond laser. Three different samples were used: NPG/SiO_2_ (red curve), intrinsic GaAs without NPG films (blue curve) and NPG/GaAs (black curve); THz waveforms measured with the intensity at (**b**) 9.28 GW/cm^2^ and (**c**) 37.14 GW/cm^2^; (**d**) The reflection of 800 nm light from the NPG films deposited on glass (red curve) and GaAs (black curve) as a function of the peak intensity of the incident femtosecond laser.

As shown in Figure 2a, we compared the THz peak to peak amplitudes from different samples, i.e., intrinsic GaAs crystal, NPG films deposited on glass slide (NPG/SiO_2_), and NPG films deposited on intrinsic GaAs substrate (NPG/GaAs). As shown in Figure 2a, the amplitude of THz pulse emitted from NPG/SiO_2_ depended on the pump intensity linearly in the low-intensity pump range below 13 GW/cm^2^ and nonlinearly in the high-intensity pump range above 13 GW/cm^2^, respectively. In the low-intensity pump region, THz field emitted from NPG/GaAs exhibited approximately the same intensity as that from NPG/SiO_2_, as shown in Figure 2b. Both THz amplitudes from NPG/GaAs and NPG/SiO_2_ were proportional to the incident laser intensity, which corroborates the optical rectification (see Figure 2a) [11,14]. THz generation differed little for NPG deposited on different substrates in the low-intensity pump region. However, as the intensity increased (>13 GW/cm^2^ in our experiments), THz generation efficiency from NPG/SiO_2_ became weaker than that from NPG/GaAs. Similar phenomenon has been observed by Kadlec et al. [12]. In the high-intensity pump region, the evanescent plasmon field on the nano-porous surface [16] of the NPG films was strong enough to accelerate free charges from air-NPG interface moving to NPG-glass interface within the pump pulse duration. The aggregation of charges resulted in an electric field opposite to the transient current, leading to attenuation of THz emission with the increase of pump intensity. On the other hand, as shown in Figure 2a, NPG/GaAs samples exhibit quite different dependence of THz emission upon the pump intensity. The THz amplitudes still scaled nearly linearly as excited by pump intensity up to 30 GW/cm^2^, above which nonlinear increase makes the THz amplitude become saturated. This indicates that the GaAs substrate could enhance the excitation of THz field from NPG films as compared with the SiO_2_ substrate. The enhancement is 1.4 times in maximum, as shown in Figure 2c. The polarization of THz electric field emitted from NPG films was observed to be parallel to the pump pulse polarization and independent of the azimuthal orientation of the samples. We monitored possible changes of the NPG films induced by intense femtosecond laser pulses by measuring the 800-nm laser reflection. Figure 2d shows the measured reflective index of 800-nm laser from the NPG films as a function of the pump intensity. As the pump laser intensity increases, the reflective index in both NPG/SiO_2_ and NPG/GaAs are approximately constant. No enhanced absorption of pump was observed under high intensity, indicating that enhanced THz emission was not related with surface or structure changes of the NPG films. The THz emission difference of NPG/GaAs and NPG/SiO_2_ should be originated from photoelectron dynamics.

**Figure 3:**
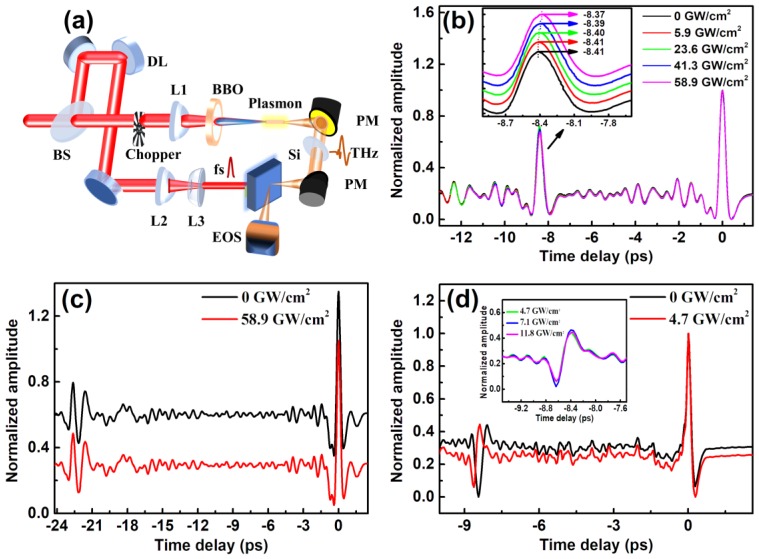
(**a**) Schematic of laser-pumped and THz-probed setup: BS, beam splitter; DL, optical delay line; L1 and L2, convex lens; L3, concave lens; BBO, β-barium borate crystal; PM, parabolic mirror; EOS, electro-optic sampling. The waveforms of THz probe pulses reflected from different samples of (**b**) NPG/GaAs, (**c**) NPG/SiO_2_ and (**d**) intrinsic GaAs were measured under different pump intensities; (**b**) The left peak of the reflected THz pulse is zoomed in the inset. Each curve was normalized and added a value of 0.2 respectively in order to distinguish imperceptible shift; (**c**) The curves were normalized. The black curve was added a value of 0.4; (**d**) The curves were normalized. The inset is the left THz pulse measured under different pump intensities.

We next explored origin of stronger THz emission from NPG/GaAs by using THz time-domain spectroscopic measurements, which are commonly used to determine the refractive index of semiconductor in THz region [17]. Those measurements enabled us to determine whether the accelerated charges were transferred into the substrates [18]. External injection of charges into the GaAs induced band-filling, bandgap shrinkage, and free-carrier absorption effects, which could produce contributions to the total change (Δn) of the refractive index [19,20,21]. For wavelengths in the transparent regime of GaAs, the band-filling and free-carrier absorption effects both contribute negative Δn, while bandgap shrinkage effect contributes positive Δn [22]. In order to observe injection of photo-induced charges from NPG films to substrates, we measured the refractive index of the substrates in THz region with the experimental setup as shown in Figure 3a. In those measurements, femtosecond laser pulses were divided into two parts by a beam-splitter with a splitting ratio of 3:1. The total pulse energy was 3 mJ. The lower energy part referred as the pump pulse was reflected into optical delay line and irradiated on the front side of sample with a spot size of ~9 mm^2^. Transmitted pulse (75% energy) from the beam-splitter was focused by a plano-convex lens of 20 cm focal length to form a filament in ambient air [23]. A frequency-doubling *β*-BBO (200 μm, type I) was inserted between lens and filament, leading to second harmonic generation. THz emission from dual-color filament [24,25] was used as the probe pulse to measure the refractive index of substrates. The THz probe pulses were focused onto the back surface of sample by a pair of parabolic mirrors. The THz probe pulse counter propagated with respect to pump pulse. The THz probe pulses were detected by the EOS method in the direction of specular reflection from back side of the sample. The temporal separation between the 800-nm pump pulse and the THz probe pulse was adjusted by a stepping motor. The 800-nm pump pulse was about 10 ps ahead of THz probe pulse. The waveforms of the THz probe pulse reflected from NPG/GaAs (see Figure 3b), NPG/SiO_2_ (see Figure 3c) and intrinsic GaAs without NPG films (see Figure 3d) were measured, respectively.

The waveforms of THz probe pulse reflected from NPG/GaAs are shown in Figure 3b under various pump intensities. There are two peaks of the reflected THz pulse in time domain. The stronger peak at 0 ps was reflected from the air-GaAs interface. The weaker THz pulse around −8.41 ps was reflected from GaAs-NPG interface. According to the time delay between two peaks, we could calculate the average refractive index of the substrate according to [26]:

(1) $$n_{\mathrm{sub}}=c\cdot \tau /d$$

where *n_sub_* is the refractive index of the substrate in THz region, and *c* speed of light in vacuum, and *τ* the time delay between two THz peaks reflected from air-GaAs and GaAs-NPG interface, and *d* the optical path difference. The corresponding time delays *τ* are 8.41, 8.41, 8.40, 8.39 and 8.37 ps under the pump intensity 0, 5.9, 23.6, 41.3, and 58.9 GW/cm^2^, respectively. As the intensity increases, the weaker reflected THz pulse moves closer to the stronger reflected THz pulse. Figure 3c,d compare the THz waveforms reflected from the substrate of NPG/SiO_2_ and NPG/GaAs, indicating that the pump laser only affected the THz refractive index of substrate GaAs in the NPG/GaAs configuration. Such a variation of the THz refraction index should be in principle induced by external injection of free charges from the NPG films [27,28]. As excited by high-intensity pump, local surface plasmon resonance is generated on the interaction area of the NPG films which causes the acceleration of free electrons. Driven by the local resonance field, the accelerated charges transport into the intrinsic GaAs which possesses a higher mobility rate than SiO_2_. As a result, less of accelerated charges assembled in the NPG films near the side of GaAs substrate as compared with that of SiO_2_ substrate. Accordingly, transfer of accumulated charges enhances THz emission under high-intensity pumps, and benefits THz generation from nano-gold films with thickness less than 100 nm.

Similar to THz emission from NPG/GaAs, we observed that the transformation of free electrons was also uncorrelated to azimuthal orientation of the GaAs substrate. As rotating azimuthal orientation, the amplitudes of the enhanced THz emission were almost the same. Interestingly, we observed that the enhanced THz emission from NPG/GaAs was related on the recovery time of electron hole pair in GaAs.

**Figure 4:**
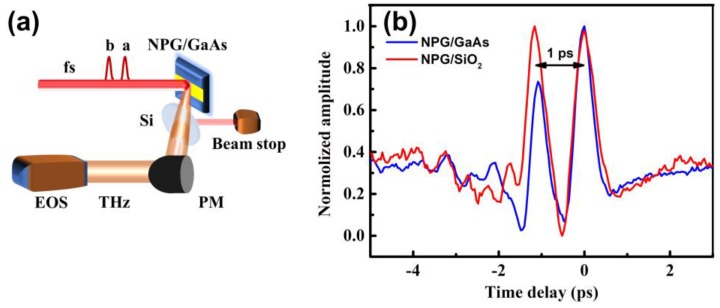
(**a**) Schematic diagram with two collinearly-propagating pulses (pulse-a and pulse-b) to irradiate on the surface of the NPG films. Pulse-a was 1 ps ahead of pulse-b. The emitted THz waveforms were detected by EOS sampling; (**b**) Normalized THz waveforms from NPG/GaAs and NPG/SiO_2_.

The third setup shown in Figure 4a was altered to study the dynamics of the charges transfer in detail. Two collinearly-propagating laser pulses, pulse-a, and pulse-b, were used to irradiate the NPG films. Pulse-a was 1 ps ahead of pulse-b. Figure 4b reveals that both pulse-a and pulse-b generated THz fields of the same peak amplitudes from the NPG/SiO_2_ sample. Though THz emission was influenced by the charges accumulation in the metallic thin films, the initial currents for THz generation excited separately by pulse-a and pulse-b were the same. It reveals that the recovery time of free charges in metallic thin films should be less than 1 ps. While in the case of NPG/GaAs, peak amplitudes of THz field excited by pulse-b is weaker than that excited by pulse-a. We assume that the accelerated charges excited by pulse-a are transferred into GaAs substrate and aggregated near the GaAs side. The dissipation time of the transferred charges which are near the GaAs side in principle depends on the recovery time of electron-hole pair in GaAs. Repulsed by the aggregated charges in GaAs, the accelerated charges excited by the subsequent pulse-b are not able to transmit into GaAs, resulting the charges accumulated in the GaAs up surface near the side of the NPG films. Eventually, the charges accumulation results in observable reduction on THz pulse emission excited by pulse-b. This gives further evidence of photoelectron injection from the NPG films to the intrinsic GaAs. In addition, the recovery time of the injected charges in the intrinsic GaAs should be large than 10 ps as confirmed in our experiments.

## 4. Conclusions

In summary, by varying the pump intensity, we have demonstrated that femtosecond laser activated optical rectification induced THz emission from NPG films with thickness of 100 nm. While under high pump intensities, THz emission from NPG/SiO_2_ was suppressed due to the accumulation of free charges in the NPG films. To enhance THz emission, we took intrinsic GaAs instead of SiO_2_ as the substrate. The accumulated charges were transferred into the GaAs substrate, which brought about observable decrease of the refractive index of GaAs in the THz region. Compared with NPG/SiO_2_, THz emission from NPG/GaAs was enhanced 1.4 times in maximum. Moreover, accumulation of charges in the GaAs substrate near NPG-GaAs interface could repulse the accelerated charges transferred from NPG films into GaAs. The resulting enhancement of THz generation could shed light on the internal photoelectrons dynamics of GaAs-deposited NPG films excited by femtosecond ultrashort pulses. Moreover, our future work will focus on design of strong THz emitters by optimizing the method of combining NPG films and semiconductors.
